# Supplementary material for: Idiopathic Infertility as a Feature of Genome Instability
Source: Life (Basel). 2021 Jun 29;11(7):628. doi: 10.3390/life11070628 (PMC8307193; doi:10.3390/life11070628)
Supplement: Supplementary file 1 [file life-11-00628-s001.zip › life-1264348-supplementary.pdf]

Supplementary Materials:

Review

Idiopathic Infertility as a Feature of Genome Instability

Agrita Puzuka [1,2], Baiba Alksere [1,3], Linda Gailite [1] and Juris Erenpreiss [1,3,*]

Table 1. The association of single-gene allelic variants with male infertility. Table 1 summarizes the latter data, including the normal function and pathological dysfunction of these genes. Genes included in the table are summarized from literature sources that were used to write this review article, the pathology name and gene functions defined using information from UniProt database https://www.uniprot.org/

Gene	Full name	Function	Pathology
AK7	Adenylate kinase 7	Nucleoside monophosphate (NMP) kinase that catalyzes the reversible transfer of the terminal phosphate group between nucleoside triphosphates and monophosphates	Multiple morphological anomalies of the flagella
AKAP3	A-Kinase anchor protein 3	Regulator of both motility- and head-associated functions such as capacitation and the acrosome reaction	Multiple morphological anomalies of the flagella
AKAP4	A-Kinase anchor protein 4	Regulator of both motility- and head-associated functions such as capacitation and the acrosome reaction	Multiple morphological anomalies of the flagella
AR	Androgen receptor	Steroid hormone receptor	Azoospermia
AURKC	Aurora kinase C	Serine/threonine-protein kinase component of the chromosomal passenger complex (CPC), a complex that acts as a key regulator of mitosis	Macrozoospermia
BDNF	Brain-derived neurotrophic factor	Influences viability, motility, nitric oxide concentration, mitochondrial activity and lipid peroxidation content	Oligo-astheno-teratozoospermia
CDKN1C	Cyclin-dependent kinase inhibitor 1C	Negative regulator of cell proliferation	Foetal growth restriction
CEP135	Centrosomal protein of 135 kDa	Microtubule binding	Multiple morphological anomalies of the flagella
CFAP43	Cilia- and flagella-associated protein 43	Sperm flagellar formation	Multiple morphological anomalies of the flagella
CFAP44	Cilia- and flagella-associated protein 44	Sperm flagellar formation	Multiple morphological anomalies of the flagella
CFAP69	Cilia- and flagella-associated protein 69	Sperm flagellar formation	Multiple morphological anomalies of the flagella

CFTR	Cystic fibrosis transmembrane conductance regulator	Vas deferens formation, reduced sperm quality	Congenital bilateral absence of the vas deferens
DCN	Decorin	Dysfunctional spermatogenesis and spermiogenesis	Azoospermia, foetal growth restriction
DLK1	Delta-like non-canonical Notch ligand 1	Inhibits adipocyte differentiation	Foetal growth restriction
DMC1	DMC1	DNA repair	Azoospermia, oligozoospermia
DNAH1	Dynein heavy chain 1, axonemal	Sperm flagellum motility	Multiple morphological anomalies of the flagella
DNMT1	DNA (cytosine-5)-methyltransferase 1	Methylation of newly formed DNA strands	Oligo-astheno-teratozoospermia
DPY19L2	Dpy-19-like 2	Attaches acrosome to the nuclear membrane	Globozoospermia
GNAS	Guanine nucleotide-binding protein (G protein), alpha-stimulating activity polypeptide	Endocrinal regulation	Oligozoospermia, hypogonadism
GRB10	Growth factor receptor-bound protein 10	Embryonic development	Foetal growth restriction
H19	Maternally inherited long non-coding RNA	Embryonic development	Foetal growth restriction
Igf2	Insulin-like growth factor 2	Regulation of cell proliferation, growth, migration, differentiation and survival	Foetal growth retardation, OAT, teratozoospermia
MAPK8IP3	Mitogen-activated protein kinase 8-interacting protein 3	Kinesin-1 transportation	Azoospermia, oligozoospermia
MEST	Mesoderm-specific transcript	Mesoderm development	Azoospermia, oligozoospermia
MLH1	MutL homolog 1	MMR	Azoospermia, oligozoospermia
MLH3	MutL homolog 3	MMR	Azoospermia, oligozoospermia
P16	Cyclin-dependent kinase inhibitor 2A	Cell cycle regulation	Oligoasthenozoospermia
PGAM5	PGAM family member 5	Mitochondrial function	Varicocele
POMC	Pro-opiomelanocortin	Opioid system regulation	Oligozoospermia, teratozoospermia
PRDM9	PR domain zinc finger protein 9	Regulating DSB positions	Azoospermia
PTPRN2	Receptor-type tyrosine-	Regulatory component in signal transduction pathway	Azoospermia,

	protein phosphatase N2		oligozoospermia
RAD51	RAD51 recombinase	DSB repair	Azoospermia, oligozoospermia
RASGRF	RAS protein-specific guanine nucleotide-releasing factor 2	Stimulates the conversion of the GDP-bound form into the active form	Globozoospermia and lack of acrosome formation
Rec8	Meiotic recombination protein REC8 homolog	Chromosome segregation at meiosis, cohesin subunit	Azoospermia
Set1	Histone-lysine N-methyltransferase	Histone H3-K4 methylation	Diminished DSB formation
SGCE	Sarcoglycan, epsilon	Subunit of sarcoglycan protein complex	Foetal growth restriction
SNRPN	Small nuclear ribonucleoprotein-associated protein N	Paternal imprinting	Foetal growth restriction
SPO11	Meiotic recombination protein SPO11	Meiotic recombination	Azoospermia, oligozoospermia
TEX11	Testis-expressed protein 11	Promotes initiation and/or maintenance of synapsis and formation of crossovers	Spermatogenic failure
TEX15	Testis-expressed protein 15	Promotes initiation and/or maintenance of synapsis and formation of crossovers	Spermatogenic failure
TKR	Transketolase-like 1	Testis-specific expression	Spermatogenic failure
TYRO3	Protein tyrosine kinase 3	Germ cell development	Spermatogenic failure
